# Duodenum Clamping Trauma Induces Significant Postoperative Intraperitoneal Adhesions on a Rat Model

**DOI:** 10.1371/journal.pone.0049673

**Published:** 2012-11-20

**Authors:** Jingrui Bai, Hongbin Liu, Donghua Li, Lihua Cui, Xianzhong Wu

**Affiliations:** 1 Graduate school of Tianjin Medical University, Tianjin, China; 2 Department of Pharmacology, Tianjin Nankai Hospital, Tianjin, China; National Cancer Institute, United States of America

## Abstract

**Objective:**

The purpose of this study was to investigate the histological and morphological changes in the first two postoperative weeks on a rat intraperitoneal adhesion model induced by duodenum clamping trauma.

**Method:**

The rat model of postoperative intraperitoneal adhesions was established in 48 male Wistar rats by laparotomy, followed by the duodenum clamping trauma. Rats were sacrificed respectively on 1^st^, 3^rd^, 5^th^, 7^th^ and 14^th^ day after the operation. The control rats were sacrificed immediately after the operation (0 day). Then the intraperitoneal adhesions were assessed macroscopically. Histopathology and immunohistochemistry were performed to evaluate the fibrosis, inflammatory responses, neovascularization, and cells infiltration in adhesion tissues. In addition, the changes of the mesothelium covering the surgical sites were examined by scanning electron microscopy.

**Results:**

Our study revealed that duodenum clamping trauma induced by mosquito hemostat can result in significant postoperative intraperitoneal adhesions formation. The extent and tenacity of intraperitoneal adhesions reached their peaks on 3^rd^ and 5^th^ days, respectively. Histopathological examination showed that all rats developed inflammatory responses at the clamped sites of duodenum, which was most prominent on 1^st^ day; the scores of fibrosis and vascular proliferation increased slowly from 3^rd^ to 5^th^ day. Myofibroblasts proliferated significantly in the adhesion tissues from 3^rd^ day, which were examined by immunohistochemical method. And the mesothelium covering the surgical sites and the adhesion tissues healed on 7^th^ day.

**Conclusion:**

This study suggests that clamping trauma to the duodenum can result in significant postoperative intraperitoneal adhesions formation, which represents an ideal rat model for intraperitoneal adhesions research and prevention. And myofibroblasts may play an important role in the forming process of intraperitoneal adhesions.

## Introduction

The intraperitoneal adhesions are pathological bonds usually be­tween the omentum, viscera and abdominal wall [1]. Etiological factors of intraperitoneal adhesions formation include peritonitis, endometriosis, radiotherapy, foreign body reaction, and so on, but the majority of intraperitoneal adhesions are caused by surgical procedures [2], [3], [4]. The incidence of intraperitoneal adhesions after operation was as high as 95% [5]. The formation of intraperitoneal adhesions is an almost inevitable complication following abdominal surgery, leading to severe clinical consequences, such as abdominal pain, adhesive small bowel obstruction and infertility [1], [2].

The intraperitoneal adhesions always took shape within the first five to seven days after the injury to peritoneum [6]. It is the result of both insufficient fibrinolytic capacity and increased fibrin formation in response to an enhanced inflammatory status of the peritoneum [3]. In recent years, many managements and drugs for adhesions prevention were applied in experimental and clinical studies, but few were proved to be really effective and safe [7], [8], [9]. Better understanding of the forming process and pathologic mechanism of intraperitoneal adhesions will contribute to the promotion of prevention measures. However, results from animal studies investigating prevention or treatment of adhesions are limited, due to lack of consistency in existing animal models. In our present model, traumatizing the duodenum by clamping with a hemostat is the direct cause of intraperitoneal adhesions. The histological and morphological changes in the first two postoperative weeks were studied and the mechanisms were investigated.

## Materials and Methods

### Experimental Animal

The animal experiment was approved by Ethics Committee of Tianjin Nankai Hospital (Permit number: SCXK-Jin-2011-0011. Tianjin, China).

Male Wistar rats, twelve-weeks-old and weighed 250–270 g, were purchased from Academy of Military Medical Sciences (Tianjin, China). Rats were housed in accordance with current national guidelines regarding animal welfare. Before the experiment, rats were kept in special-pathogen-free conditions for one week, with standard laboratory chow and water available *ad libitum*. The environment was maintained at 18–26°C with a relative humidity of 30–70% and in a 12 hours light/12 hours dark cycle.

### Surgical Procedure

**Figure 1:**
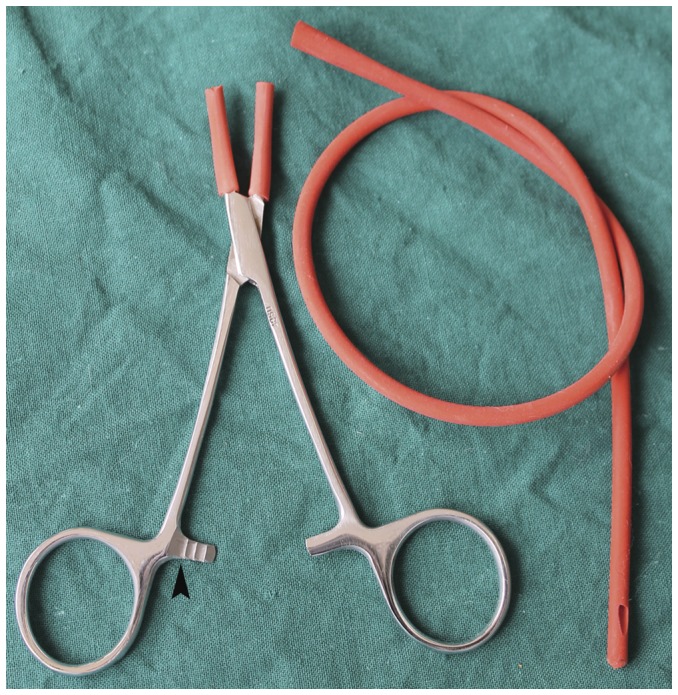
The special devise for the operation. A mosquito hemostat with curved tips and full teeth, which were shielded by a segment of rubber single Nelaton Catheter. (Black arrowhead: the second ratchet of the hemostat).

The key surgical instrument for the model establishment is a mosquito hemostat with curved tips and full teeth (Jinzhong, Shanghai, China), which is 12.5 cm in length and has three ratchets. The teeth of the mosquito hemostat were shielded by a segment of a 12Fr rubber single Nelaton Catheter (Welllead, Guangzhou, Guangdong, China) (Figure 1). The mean value of clamping force by the second ratchet is 18.3±0.9 N, which was detected by electronic universal testing machine (HTE Model, Hounsfield Company, Surrey, England).

After fasting overnight before the operation, all animals were assigned randomly to six groups, 8 in each. All surgical procedures were performed by the initial under sterile conditions. Animals were anesthetized by intraperitoneal injection with 10% Chloral Hydrate solution (3 ml/kg bodyweight; Tianjin Chemical Reagent Company, Tianjin, China), after which the abdomen was shaved and swabbed with 75% alcohol. Then the prepared rat was fixed in the supine position and the abdomen was covered with a fenestrated sterile drape. Heat loss was prevented by placing the rat on a warming tray together with a hot incandescent light during the operation. An up midline incision was made to identify and expose the duodenum. Subsequently, from the pylorus down, grasp the duodenum in the clamp of the hemostat and squeeze the handle of hemostat to the second ratchet for 3 seconds. There were nine segmental clamping in total and at intervals of 0.5 cm. Then, the injured bowel was return into the abdominal cavity gently, and Bupivacaine (0.25%, 1.5 ml; Mintong, Zhuhai, China) was infiltrated into the abdominal wound for postoperative analgesia. Finally, close the peritoneum, fasciae and abdominal musculature applying simple running sutures, and close the skin applying simple interrupted sutures, using 4–0 silk nonabsorbable surgical suture (Cangsong, Shanghai, China) [10]. All procedures were finished with 10 minutes.

Postoperatively, the rats were transferred to individual cages, given O_2_ by mask and infrared warming lamp irradiation until they were awake and moving. Rats were fasted for 24 hours, but free to water. No other analgesic and antibiotic drugs were given after the operation.

### Macroscopical Evaluations

The animals were sacrificed by cervical dislocation after the surgery (0 day), and then on 1^st^, 3^rd^, 5^th^, 7^th^ and 14^th^ day. The abdominal cavity was opened in U-shaped incision and firstly examined for the incidence of intraperitoneal adhesions. The extent and tenacity of the adhesions were graded by two pathologists in a blinded fashion, using two different scoring systems that were respectively described by Nair [11] and Zuhlke [12] (Table 1).

**Table 1:** Scales for macroscopical evaluations.

Score	Extent [11]	Tenacity [12]
0	No adhesions.	No adhesions.
1	Single band of adhesion between viscera or from one viscus to theabdominal wall.	Filmy adhesion, easy to separate by blunt dissection.
2	Two bands, either between viscera or from viscera to the abdominal wall.	Strong adhesion, blunt dissection possible, partly sharp dissection.
3	More than two bands between viscera or from viscera to the abdominal wall.	Stronger adhesion; sharp dissection necessary.
4	Multiple dense adhesions or viscera directly adherent to the abdominal wall,irrespective of number and extent of adhesive bands.	Very strong adhesion between organs; its division by sharpdissection damages organ serosa.

### Histological Evaluations

After macroscopical evaluations, specimens of injured duodenum containing adhesion tissues were excised, rinsed with 0.9% Normal Saline (Otsuka, Guangdong, China) and then carefully dissected into two parts. One was for light microscopy; the other was for scanning electron microscopy (SEM).

For light microscopy examination, the samples were immersed in 10% formalin solution (Tianjin Chemical Reagent Company, Tianjin, China) for 24 hours and dehydrated in a graded series of ethanol before embedded in paraffin. Serial sections were stained with Hematoxylin and Eosin (H&E). Each slide was selected five random visual fields (×200) and scored using the following scales (Table 2) [13], [14], [15], to evaluate the grade of fibrosis, inflammation and neovascularization of adhesion tissues. Immunohistochemistry staining of pan cytokeratin (PCK), vimentin (Vim) and α-smooth muscle actin (α-SMA) was carried out to evaluate the cellular infiltration in the forming process of adhesions (monoclonal mouse antibody, 1∶200 dilution; labeled Streptavidin/Peroxidase biotin method, Boster, Wuhan, China), and the control sections were treated with phosphate buffered saline (PBS, PH = 7.0) rather than any of the first antibodies. The fibroblast was positive for PCK and Vim, but negative for α-SMA; nevertheless the myofibroblast stained positive for Vim and α-SMA, and negative for PCK.

**Table 2:** Scales for histological (H&E) evaluations [13], [14], [15].

Scale	Fibrosis	Inflammation	Microvessel density
0	None	None	None
1	Slight	Giant cell or foreign body-macrophages, lymphocytes and plasma cells.	1–3 capillaries
2	Moderate	As score 1 plus polymorphonuclear granulocytes and eosinophils.	4–10 capillaries
3	Severe	Abundant inflammatory cells and microabscesses.	More than 10 capillaries.

All slides were evaluated by two pathologists in a blinded manner with light microscope (LEICA DM4000B, LAS Version 3.7.0, Germany). Image-Pro® Plus v 6.0 For Windows (Media Cybernetics, Silver Spring, Maryland, USA) was also used in the evaluations of Immunohistochemistry staining to account the value of integral optical density of the target areas.

For SEM, the specimens were fixed with 2.5% glutaraldehyde (Sigma-Aldrich, Saint Louis, USA) in PBS for 4 hours, dehydrated in increasing alcohol series, critical point dried (CPD-030, BAL-TEC, Switzerland), sputter coated with gold ion (SCD-005, BAL-TEC, Switzerland). Examination and photographs were obtained with scanning electron microscope (FEI Quanta 200, FEI, and Holland).

### Statistics

Macroscopical and histological scores of intraperitoneal adhesions were expressed as mean±SD, and analysed by one-way ANOVA with post-hoc Bonferroni tests. A *P*-value<0.05 was considered statistically significant. Analysis was performed using IBM SPSS Statistics version 19 (IBM SPSS, Chicago, Illinois, USA).

## Results

**Figure 2:**
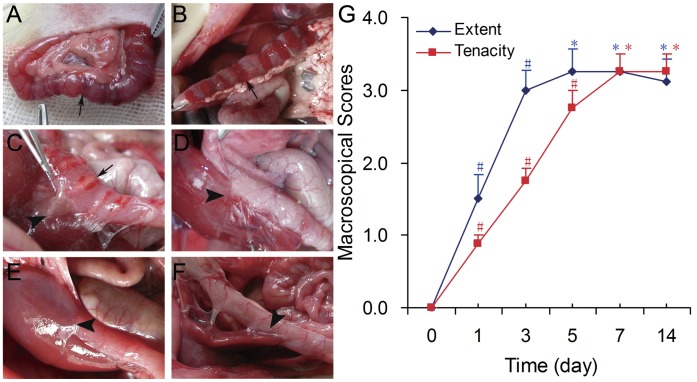
Representative macroscopical photos and adhesion scores. A–F) Macroscopical photos of 0 day, 1^st^, 3^rd^, 5^th^, 7^th^ and 14^th^ day. G) Mean of adhesion scores by macroscopical evaluations in all groups. n = 8, #: *P*<0.05, *: *P*>0.05. Vs. preceding group. (Surgical site: arrow; adhesion: arrowhead).

Macroscopically, no adhesion was found in all eight rats on 0 day. In other five groups, the incidence of adhesions was 87.5% (7/8) for 1^st^ day, 100% (8/8) for 3^rd^ day, 5^th^ day, 7^th^ day and 14^th^ day. The surgical sites were oncotic and hemorrhagic in the first three days; fibrin appeared and deposited around the injured on 1^st^ day, and filmy adhesions were detected on all rats on 3^rd^ day. The intraperitoneal adhesions always formed among the duodenum, liver, omentum, even the stomach, diaphragma and abdominal wall. The extent of adhesions continuingly expanded till 3^rd^ postoperative day, whereas the tenacity kept strengthening till 5^th^ day (Figure 2).

**Figure 3:**
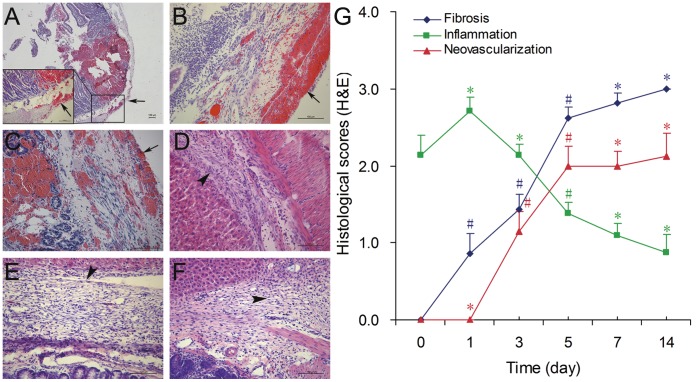
Histological micrographs and scores of fibrosis, inflammation and neovascularization (H&E). A) Micrograph of 0 day (×50, lower left corner; ×200, bar = 100 µm); B–F) Micrographs of 1^st^, 3^rd^, 5^th^, 7^th^ and 14^th^ day (×200, bar = 100 µm. Surgical site: arrow; adhesion: arrowhead). G) Mean of fibrosis, inflammation and neovascularization scores by histological evaluations in all groups. #: *P*<0.05, *: *P*>0.05. Vs. preceding group.

Under light microscope, the mucosa, submucosa and even smooth muscle of the surgical intestine were damaged by the clamping of the hemostat (0 day). On 1^st^ day, there was marked inflammatory cell infiltration in the injured tissues; and the number of these inflammatory cells decreased from 3^rd^ day till 7^th^ day. The fibrosis of the adhesion tissues developed over time, especially from 3^rd^ day to 5^th^ day after the operation. The neoformative vessels appeared in the adhesion tissues on 3^rd^ day, and its number increased slightly in the following days (Figure 3).

**Figure 4:**
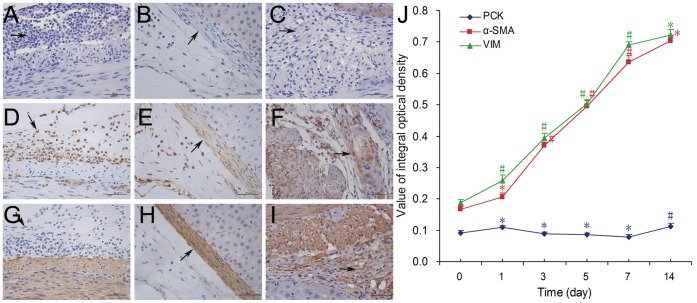
Micrographs of immunohistochemistry staining and the expression of PCK, Vim and α-SMA of 0, 7^th^ and 14^th^ day. Representative immunohistochemistry staining images stained with PCK (A–C), Vim (D–F) and α-SMA (G–I) antibody of 0 day (A, D, G), 7^th^ day (B, E, H), 14^th^ day (C, F, I) (×400, bar = 50 µm, target area: arrow). J) The values of integral optical density for PCK, Vim and α-SMA of 0 7^th^ and 14^th^ day. #: *P*<0.05, *: *P*>0.05. Vs. preceding group.

The value of integral optical density of the target areas of Immunohistochemistry staining micrographs were quantified and analyzed by Image-Pro® Plus v6.0 For Windows (Figure 4). PCK was not detected in almost all the samples. Surprisingly, Vim and α-SMA were positive in the fibroblast-like cells with spindle nucleus in adhesion tissues; and their mean value of integral optical density kept increasing till 7^th^ day (Figure 4).

**Figure 5:**
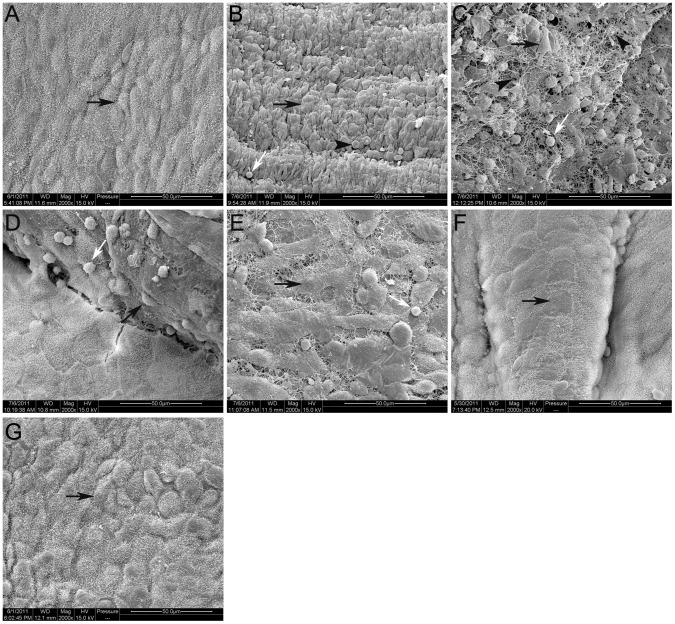
SEM images of mesothelium covering the duodenum and adhesion tissues. A) Normal control from additional rats. B–G) Micrographs showed the destruction and regeneration of the mesothelium on 0 day, 1^st^, 3^rd^, 5^th^, 7^th^ and 14^th^ day (×2000, bar = 50 µm. Mesothelial cell: black arrow; RBC: black arrowhead; inflammatory cell: white arrow).

The destruction and regeneration of peritoneal mesothelium covering the surgical sites were clearly demonstrated by SEM imagines (Figure 5). The normal visceral peritoneum covering the duodenum was composed of many flat mesothelial cells. These cells overlapped with each other tightly and with lots of microvilli on their surfaces. The mesothelium on the surgical sites was broken by the clamping trauma on 0 day; the mesothelial cells were swollen and deformed, the microvilli on which were disappeared; the cell junctions were lost and the basement membrane was exposed. Inflammatory cells including macrophages, and red blood cells (RBC) leaked out immediately after the operation. On 1^st^ day, these transudatory cells were enwrapped by deposited fibrin; and blood clots were formed to stop bleeding. Then, on 3^rd^ day, the injured sites adhered to the surrounding organs by fibroin net. The mesothelial cells, with sparse and short microvilli, began to proliferate from the edge of the injury; lots of inflammatory cells and few RBC could also be seen on the injured surface. On 5^th^ day, most parts of the surgical sites were re-covered by mesothelial cells, which overlapped with each other loosely, and with very short microvilli. On 7^th^ day, the mesothelial cells completely overlaid the surgical sites and the adhesion tissues; and on 14^th^ day, the number and length of the microvilli improved significantly, which were rehabilitated to the normal condition.

In summary, the extent and tenacity of intro-peritoneal adhesions reached their peaks on 3^rd^ and 5^th^ days, respectively. The inflammation of the adhesion tissues was most serious on 1^st^ day; the fibrosis and the neovascularization developed slowly from 3^rd^ to 5^th^ day. Myofibroblasts proliferated significantly in the adhesion tissues from 3^rd^ day, which were examined by immunohistochemical method. And the mesothelium covering the surgical sites and the adhesion tissues healed on 7^th^ day.

## Discussion

The intraperitoneal adhesions following abdominal surgery remains an ongoing challenge, without ideal products or measures for adhesions reduction. A reliable animal model that allows for objective quantification of adhesions is a key component in elucidating the authenticity of various anti-adhesive strategies [16]. This study evaluated a novel animal model of intraperitoneal adhesions, and suggested this rat model induced by duodenum clamping trauma to be consistent, reliable and reproducible.

Methods of intraperitoneal adhesion model include abdominal sidewall defect, cecal abrasion, peritoneal excision or abrasion, uterine horn injuries, and so on [15], but the results are inconsistent. Many proposed adhesion models were criticized because of observer bias and the subjective nature of recording the adhesions characteristics. For example, Gaertner *et al.* evaluated the conventional sidewall models involving cecal abrasion and peritoneal excision or abrasion, and found the classical sidewall models showed inconsistent patterns of adhesions formation and were difficult to evaluate [17]. In addition, although Whang *et al.* suggested the peritoneal button technique to be a most consistent and reproducible technique for intraperitoneal adhesion model [16]; some researchers criticized this approach, claiming that the peritoneal button technique leads to exaggerated results when evaluating anti-adhesive measures [18]. Therefore, it is imprecise to deduce definitive conclusions from anti-adhesive proposals if no credible adhesion model is available. A readily reproducible adhesion model will ensure better evaluation of the anti-adhesive measures, because confounding results from the model will be minimized.

In this study, we developed an intraperitoneal adhesion model induced by duodenum clamping trauma. We shielded the hemostat teeth with rubber single lumen Nelaton Catheter to avoid the injury caused by direct collision between the metal teeth, which can lead the injured duodenum to necrosis. Therefore, with this new devise, on the one hand, clamping the duodenum can lead to adequate injury both to duodenal serosa and inner-lumen mucosa, which will result in intraperitoneal adhesions; on the other hand, this clamping trauma is moderate and the model animal can survive with the formation of adhesions. Although the classical sidewall models involving cecal abrasion and peritoneal excision or abrasion popularly used by many investigators [19], it was impossible to accurately control the damage given to each animal, because the area and degree excised or abrased are difficult to keep consistent in all animals, and the adhesions can not be scored exactly [17]. However, on the duodenum clamping trauma rat model, the adhesions always formed among the duodenum, liver and omentum, which were more stable and easily to score because of the standardized and controllable surgical procedures.

In addition, adhesions secondary to operations in upper abdominal region occurred more frequently in recent years, due to the increase of surgical treatments on diseases of liver, gall and pancreas [20]. But most of the previous adhesion models focus on adhesions in lower abdominal region and pelvic cavity, such as the classical sidewall defect model and the uterine horn injuries model. The rat model we recommended is accurately located on the duodenum, which imitates the adhesions in the upper abdominal region.

The peritoneal mesothelium is a highly specialized monolayer of polarized flat epithelial cells that covering the entire surface of the abdominal cavity. It serves as a protective anatomical barrier, as a non-adhesive frictionless interface for the movement of abdominal organs and is involved in the formation and turnover of abdominal fluid [21], [22]. Injury to the peritoneum is often associated with structural and functional alterations of the mesothelium, which may result in peritoneal healing and adhesions formation [23]. Histopathogenesis of inflammation and repair of the mesothelium are involved in the process of adhesions formation. Moreover, postoperative peritoneal adhesions are considered as a consequence of redundant fibrin formation and insufficient fibrinolytic activity in response to enhanced inflammatory status revoked by peritoneal impairment [24]. Under normal conditions, the generation and degradation of fibrin could be a dynamic balance, which was broken under pathological conditions [25].

In our present model, the damage to the duodenum was accurately located and carried out by hemostat-clamping, leading to the injured sites congestive and swollen when the operation was finished. Local inflammatory response was triggered in the clamping sites, resulting in fibrin-rich exudates formed nearby [26]. Simultaneously, the mesothelial monolayer covering the duodenum was damaged seriously. The mesothelial cells lost their original form and cell junctions, and infiltrated by inflammatory cells on 1^st^ postoperative day, which was confirmed by diZerega [27]. The fibrin deposited on the damaged areas contributing to hemostasis and tissue repair of the injury. Nevertheless, if not degradated by 3^rd^ day, the deposited fibrin became filmy adhesion tissues connecting the injured duodenum and the adjacent organs or tissues, such as the liver, the omentum and the abdominal wall. Some people believed that adhesions formation occurred when two injured peritoneal surfaces were apposed [27], [28]. However, in this new adhesion model, adhesions formed between the injured duodenum and the surrounding tissues, which were protected in the operation. There were two possibilities about this phenomenon: the first one is the surfaces of the adjacent tissues are destroyed by the local inflammation; the second one is that the adhesions form once one of the apposed surfaces is injured.

From 1^st^ postoperative day, lots of fibroblast-like cells with spindle nucleus intruded into the deposited fibrin and proliferated, bringing about more extracellular matrix (ECM) including collagen [27]. New blood vessels appeared in adhesion tissues from 3^rd^ postoperative day [29]. At the same time, the range of adhesions enlarged very slowly; in contrast, the strength needed to separate the adhesions increased greatly. On the SEM photomicrograph of 1^st^ day, various inflammation cells appeared on the injured surface, simultaneously with a lot of elongated, flattened, irregularly shaped cells [30], [31]. These mesothelial cells, which connected with each other loosely, covered most part of the injured surface on 5^th^ day; but their microvilli were still very sparse and short.

On 7^th^ day, the adhesions were too strong to be separated by blunt dissection. The adhesion tissues were full of fibroblast-like cells [27]. New vessels of different diameters presented. The number of mesothelial cells increased significantly compared with 5^th^ day; they completely overlaid the injured surface, regularly and tightly, although the microvilli on which were still very short. On 14^th^ day, the macroscopical and histological evaluations changed little compared to 7^th^ day, except that the microvilli of the mesothelial cells were more and longer, similar to the normal [31], [32].

Here we got a question: what were the fibroblast-like cells in the adhesion tissues? For a long time, fibroblasts were considered to be the main cells that secrete fibrin and transform deposited fibrin into fibrous, permanent adhesions [27], [32], [33], [34]. In our experiment, immunohistochemistry staining of PCK, Vim and α-SMA was employed to validate this hypothesis. However, the results showed that it’s myofibroblasts who proliferated prominently in the adhesion tissues.

In fact, the myofibroblast is a special form of fibroblast, characteristically expressing α-SMA^+^and acquisition of contractile features. It can be differentiated form multiple sources, such as local primary producers, epithelial cells, mesenchymal cells and endothelial cells, when stimulated by cytokines and mechanical tension in wound healing [35], [36], [37]. During the acute inflammation period after operation, a variety of cytokines and chemotactic factors were secreted into the ECM. Myofibroblast progenitors proliferated and migrated within provisional matrix of the wound clot containing the platelet-derived growth factor, and transformed into myofibroblasts by transforming growth factor beta, which could induce myofibroblasts decreasing by apoptosis in normal wound healing [38], [39], [40]. However, under many pathological situations, myofibroblasts persisted and continued to remodel the ECM by synthesizing ECM components such as collagen types I and III, resulted in adhesions formation or even fibrosis of organs [41]. And in patients after surgery, postoperative complications such as abdominal pain, vomiting, and adhesive illus may relate to the contractile feature of myofibroblast.

In conclusion, clamping trauma to the duodenum can induce significant postoperative intraperitoneal adhesions formation, which represents an ideal animal model for intraperitoneal adhesions research and prevention. And myofibroblasts may play an important role in the forming process.
